# Bermuda grass latent virus in Australia: genome sequence, sequence variation, and new hosts

**DOI:** 10.1007/s00705-022-05434-6

**Published:** 2022-04-08

**Authors:** Nga T. Tran, Ai Chin Teo, Kathleen S. Crew, John E. Thomas, Paul R. Campbell, Andrew D. W. Geering

**Affiliations:** 1 Queensland Alliance for Agriculture and Food Innovation, Centre for Horticultural Science, Ecosciences Precinct, The University of Queensland, GPO Box 267, Brisbane, QLD 4001 Australia; 2 Department of Agriculture and Fisheries, Ecosciences Precinct, GPO Box 267, Brisbane, QLD 4001 Australia

## Abstract

Bermuda grass latent virus (BGLV; genus *Panicovirus*) is identified for the first time in Australia and in only the second country after the USA. A full-length genome sequence was obtained, which has 97% nucleotide sequence identity to that of the species exemplar isolate. Surveys for BGLV, utilising a newly designed universal panicovirus RT-PCR assay for diagnosis, demonstrated widespread infection by this virus in a broad variety of Bermuda grass cultivars (*Cynodon dactylon* and *C. dactylon* × *C. transvaalensis*) grown in both New South Wales and Queensland. The virus was also detected in Rhodes grass (*Chloris gayana*) and Kikuyu grass (*Cenchrus clandestinus*), which are both important pasture grasses in subtropical Australia, and the latter is also grown as turf. Furthermore, the Rhodes grass plant, which had strong mosaic symptoms, was also infected with sugarcane mosaic virus, warranting further investigations as to whether synergistic interactions occur between these two viruses.

**Supplementary Information:**

The online version contains supplementary material available at 10.1007/s00705-022-05434-6.

Bermuda grass (*Cynodon dactylon* and *C. dactylon* × *Cynodon transvaalensis* hybrids) is among the most widely planted turf grass species in warm temperate and subtropical regions of Australia [2, 9, 11]. Bermuda grass latent virus (BGLV) is a recently recognized virus from Bermuda grass in the USA that has tentatively been classified as a member of the genus *Panicovirus*, family *Tombusviridae* [15]. As virions were observed in both symptomatic (chlorosis) and asymptomatic plants, a correlation between the presence of the virus and symptoms could not be established, and the infection was therefore considered to be latent [15]. However, any perceptions about the lack of economic importance of BGLV have changed with the emergence of a new and devastating disease of St. Augustine grass (*Stenotaphrum secundatum*) ‘Floratam’ in central and southern Florida called viral lethal necrosis. Viral lethal necrosis is thought to be due to a synergistic interaction between a panicovirus [1], which was later identified as BGLV (P. Harmon, 2019, pers. com.), and sugarcane mosaic virus (SCMV), a member of the genus *Potyvirus*. When either virus is present as a single infection, the symptoms are mild and not reason for major concern [12, 15].

SCMV has recently been recorded as a pathogen of St. Augustine grass in Australia [17] but BGLV has yet to be found in any plant species. However, given the long history of cultivation of Bermuda grass in Australia, often using cultivars imported from the USA, it seems likely that BGLV could have been inadvertently introduced into the country, especially as plants can be asymptomatically infected and therefore escape the notice of plant quarantine officers. In this paper, we describe surveys done to search for BGLV in Australia, resulting in detections in several clonal accessions of Bermuda grass, as well as in Rhodes grass (*Chloris gayana*) and Kikuyu grass (*Cenchrus clandestinus*). The complete genome sequence of an Australian isolate of BGLV is also presented.

In February 2020, a weedy Bermuda grass plant with chlorotic mottling and spindle-shaped lesions on the leaves (Fig. 1a), was observed beside a road on a turf farm in south-east Queensland. A stolon from this plant was collected and planted in a pot in an insect-free glasshouse at the Ecosciences Precinct, Dutton Park, Queensland. Species identification of this plant was confirmed by DNA barcoding of the chloroplast maturase K gene (*matK*) using primers *matK*472F and *matK*1248R [19], and the DNA sequence was deposited in the GenBank database (Table 1). The symptoms disappeared when the plant was grown in the glasshouse. Leaves from this plant were freeze-dried and lodged in the Queensland Department of Agriculture and Fisheries (QDAF) Plant Virus Collection under isolate number 5657. A small-scale, crude virus purification was done using the virus miniprep method of Geering *et al.* [3]. Carbon-stabilised, necoloidine-coated grids were floated on drops of the virus miniprep and then negatively contrasted with 1% ammonium molybdate, pH 5.8, for viewing under a JEOL JEM-1400 transmission electron microscope. Isometric virions with a modal diameter of 27 nm were observed (Fig. 1d), suggesting infection with a panicovirus.

**Fig. 1:**
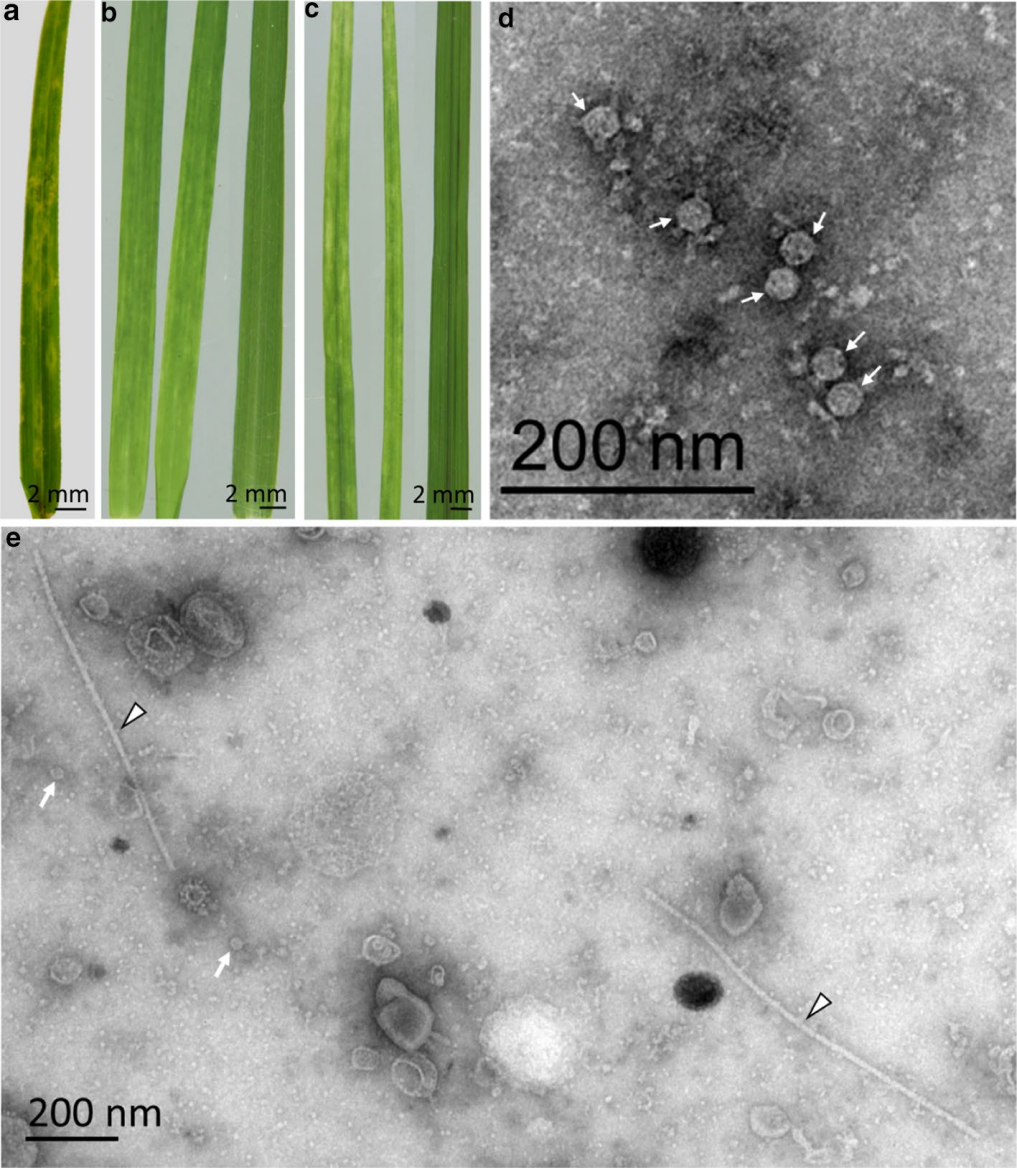
(**a**) Leaf of a common Bermuda grass (*Cynodon dactylon*) plant (virus isolate 5657) with spindle-shaped lesions, (**b**) leaves of a Bermuda grass ‘Gully Gold’ plant (virus isolate 5663) displaying mild mosaic symptoms (left and centre), as opposed to an asymptomatic leaf (right) from a common Bermuda grass plant), (**c**) leaves of a Rhodes grass (*Chloris gayana*) plant (virus isolate 5651) displaying mosaic symptoms (left and centre), as opposed to an asymptomatic leaf from an uninfected plant of the same species (right), (**d**) isometric virions (arrows) of Bermuda grass latent virus (BGLV) observed in a leaf extract from virus isolate 5657, and (**e**) two types of virion, isometric (BGLV, arrows) and flexuous and filamentous (sugarcane mosaic virus, arrow heads), observed in a leaf extract from virus isolate 5651

**Table 1:** Survey results for Bermuda grass latent virus on grasses collected from New South Wales (NSW) and Queensland (QLD), Australia

Species	Cultivar/common name	BGLV	Nearest town	State	Collection date (dd/mm/yyyy)	Isolate accession ^a^	GenBank accession no.		
							BGLV^b^	SCMV^c^	*matK*^d^
*Cynodon dactylon*	‘Wintergreen’	−	Carlingford	NSW	23/07/2018	…	…	…	…
	‘Gully Gold’	+	Windsor	NSW	08/04/2019	5663	MZ671028	…	…
	‘Greenlees Park’	+	Windsor	NSW	08/04/2019	5660	MZ671025	…	…
	‘Greenlees Park’	−	Cherrybrook	NSW	08/04/2019	…	…	…	…
	‘Grand Prix’	−	Pitt Town Bottoms	NSW	10/04/2019	…	…	…	…
	‘Windsor Green’	+	Pitt Town Bottoms	NSW	10/04/2019	5659	MZ671024	…	…
	‘Windsor Green’	−	Cornwallis	NSW	08/04/2019	…	…	…	…
	‘CT-2’	−	Cornwallis	NSW	08/04/2019	…	…	…	…
	‘C1’ (Legend^®^)	−	Cornwallis	NSW	08/04/2019	…	…	…	…
	‘C1’ (Legend^®^)	−	Redlands	QLD	18/04/2019	…	…	…	…
	‘Oz-E-Green’ (Oz Tuff Turf^®^)	−	Redlands	QLD	18/04/2019	…	…	…	…
	‘Oz-E-Green’ (Oz Tuff Turf^®^)	−	Rathdowney	QLD	04/10/2018	…	…	…	…
	‘Oz-E-Green’ (Oz Tuff Turf^®^)	−	Boyland	QLD	18/04/2018	…	…	…	…
	‘Wintergreen’	+	Boyland	QLD	24/10/2018	5661	MZ671026	…	…
	‘Plateau’	−	Indooroopilly	QLD	14/10/2018	…	…	…	…
	Common Bermuda grass	+	Chambers Flat	QLD	11/02/2020	5657	MZ671022	…	OL456263
	Common Bermuda grass	+	Rocklea	QLD	23/08/2021	5812	OK258314	…	OL456264
	Common Bermuda grass	+	Rocklea	QLD	23/08/2021	5815	OK258317	…	OL456267
	Common Bermuda grass	+	Rocklea	QLD	23/08/2021	5816	OK258318	…	OL456268
*Cynodon* sp*.*	UQ 545	−	Redlands	QLD	18/04/2018	…	…	…	…
*C. dactylon* × *C. transvaalensis*	‘Santa Ana’	−	Freemans Reach	NSW	08/04/2019	…	…	…	…
	‘DT-1’ (TifTuf^®^)	+	Theresa Park	NSW	14/10/2021	5818	OL539730	…	…
	‘DT-1’ (TifTuf^®^)	−	Allenview	QLD	17/08/2018	…	…	…	…
	‘Tift94’ (TifSport^TM^)	+	Wamuran	QLD	23/07/2018	5658	MZ671023	…	…
	‘Tift94’ (TifSport^TM^)	+	Redlands	QLD	18/04/2019	5662	MZ671027	…	…
	Latitude 36 Bermuda grass	−	Redlands	QLD	18/04/2019	…	…	…	…
	‘Tifway’	−	Yeppoon	QLD	13/12/2018	…	…	…	…
*Chloris gayana*	Rhodes grass	+	Rocklea	QLD	01/03/2021	5651	MZ852894	OK258313	OL456262
*Cenchrus clandestinus*	Kikuyu grass	+	Rocklea	QLD	23/08/2021	5813	OK258315	…	OL456265
	Kikuyu grass	+	Rocklea	QLD	23/08/2021	5814	OK258316	…	OL456266

^a^Queensland Department of Agriculture and Fisheries Plant Virus Collection

^b^BGLV = Bermuda grass latent virus

^c^SCMV = sugarcane mosaic virus

^d^*matK* = chloroplast maturase K gene of the host

To test the hypothesis that the plant was infected with a panicovirus, reverse transcription PCR (RT-PCR) and amplicon sequencing were done. In the absence of a proper diagnostic control and adequate knowledge of sequence diversity, we chose to develop a universal panicovirus assay. RNA-dependent RNA polymerase (RdRp) amino acid sequences of the exemplar isolates of BGLV (GenBank KX758441) and members of the three officially recognized panicovirus species, *Panicum mosaic virus* (GenBank U55002), *Cocksfoot mild mosaic virus* (GenBank EU081018), and *Thin paspalum asymptomatic virus* (GenBank JX848617), were aligned using the MUSCLE algorithm within the MEGA software package, and the aligned sequences were back-translated to give the DNA sequence alignment. Potential primer-binding sites with minimal degeneracy were identified using GPrime [6]. Two degenerate PCR primers were designed, PanicoRdRp-F (5ʹ TGCGCWATWGGATTYGAYGC 3ʹ) and PanicoRdRp-R (5ʹ CCTCATCATCCWCCGAGCA 3ʹ), which were predicted to produce an amplicon of *c*. 350 bp. RT-PCR primer annealing temperatures were optimised using Panicum mosaic virus (PMV) isolate 2349 [16] from the QDAF Plant Virus Collection, and this virus was used as a positive diagnostic control.

Total nucleic acid extracts (TNAEs) were prepared using a BioSprint 15 DNA Plant Kit (catalog number: 941517, QIAGEN) as per the manufacturer’s instructions but omitting the RNase A digestion step. RT-PCR assays were done using a MyTaq One-Step RT-PCR Kit (catalog number: BIO-65049, Bioline Meridian Bioscience) as per the manufacturer’s instructions. RT-PCR thermocycling conditions were 20 min at 45°C and 1 min at 95°C; followed by 40 cycles of 95°C for 10 s, 65°C for 10 s, and 72°C for 30 s; and finally, 2 min at 72°C. The amplicon was electrophoresed through a 1.5% (w/v) agarose gel and visualised on a UV transilluminator after staining with ethidium bromide. Virus isolate 5657 was RT-PCR positive. Amplicons were sequenced directly by Macrogen Inc., South Korea, and BLASTn and BLASTx search results showed that BGLV (GenBank KX758441) was the closest match, with 96.5% and 100% identity at the nt and amino acid (aa) levels, respectively.

To better characterise virus isolate 5657, high-throughput sequencing was done. Total RNA was extracted using a TRIzol Plus RNA Purification Kit (catalog number: 12183555, Invitrogen) as per the manufacturer’s instructions. The total RNA extracts were submitted to the Australian Genome Research Facility (AGRF; Melbourne, Australia) for library preparation and sequencing. Ribosomal RNA was removed using a TruSeq Stranded Total RNA with Ribo‐Zero Plant kit (Illumina) with 500 ng of input RNA. The library was sequenced as 100-bp paired-end reads on an Illumina NovaSeq platform with an SP 300 cycle kit. The sequences were paired, and adaptor and primer sequences were removed using the BBDuk Plugin for Geneious Prime v. 2021.0.1 (Biomatters Ltd., Auckland). Trimming options included removal of reads with a Phred value less than 30 and shorter than 30 nt in length. One million reads, which were randomly sampled from 110,832,104 reads obtained after trimming for quality, were assembled *de novo* using CLC Genomics Workbench v. 6.5 (QIAGEN) with automatic word size and bubble size and a minimum contig length of 700 nt. The number of contigs produced after *de novo* assembly was 239 and these were sorted by length and sequence homologies identified by doing a BLASTn search of the NCBI Nucleotide Database. One contig of 4,097 nt had a significant hit to BGLV but no other viruses were identified. The sequence of the exemplar isolate of BGLV (GenBank KX758441) was then used as a scaffold to map all reads, producing a contig of 4,044 nt, which was assembled from 78,263,232 reads, with an average depth of coverage of 1,580,041 sequence reads.

The 5ʹ terminus of the genome of isolate 5657 was determined using a 5ʹ/3ʹ RACE Kit, 2nd Generation (catalog number: 03353621001, Roche Applied Science) as per the manufacturer’s instructions and with the virus-specific, reverse primers listed in Supplementary Table S1. The 3ʹ end was confirmed by polyadenylating the RNA using *Escherichia coli* poly(A) polymerase (catalog number: M0276S, New England Biolabs). cDNA of the polyadenylated RNA was synthesised using Superscript IV reverse transcriptase (catalog number: 18090010, Invitrogen) and an oligo (dT) primer (5ʹ CACGGATCCCGGG(T)_17_V 3ʹ) based on potyvirid primer 1 of Gibbs and Mackenzie [5]. PCR was then done using MyTaq HS Red DNA Polymerase (catalog number: BIO-21115, Bioline Meridian Bioscience) using the same oligo (dT) primer, paired with a virus-specific forward primer (Supplementary Table S1). PCR amplicons obtained from 5ʹ and 3ʹ RACE were sequenced directly by Macrogen Inc., South Korea.

The genome sequence of virus isolate 5657 (GenBank MZ671022) contains 4,047 nt and is 97% identical to the exemplar isolate of BGLV (GenBank KX758441). The 5ʹ UTR of virus isolate 5657 is 3 nt longer than that of the exemplar isolate, but otherwise, its genome architecture (Supplementary Fig. S1) is identical to that previously described [15], which in turn was deduced from comparisons with PMV [20, 21].

To survey for BGLV, a collection of 23 accessions of Bermuda grass representing 16 cultivars was assembled from a variety of sources (Table 1) and grown in the glasshouse at the Ecosciences Precinct, Dutton Park, Queensland. TNAEs were prepared using a BioSprint 15 DNA Plant Kit. Diagnoses were done using the universal panicovirus RT-PCR assay described above. Eight accessions returned positive results, and the sequenced amplicons all matched BGLV following BLASTn searches. These accessions originated from both New South Wales and Queensland (Table 1). The sequences were aligned, and pairwise sequence comparisons were done using Geneious Alignment in Geneious Prime, and all had 87.4–97.0% nt sequence identity to the exemplar isolate of BGLV but less than 68.1% nt sequence identity to the exemplar isolates of the other three recognized panicovirus species (Supplementary Table S2). BGLV was detected in two accessions of ‘Tift94’ that were sourced from different locations in south-east Queensland, one from a turf farm at Wamuran and the second from a turf research facility at Redlands (Table 1). ‘Tift94’ was bred in the USA and is a sterile hybrid Bermuda grass, which can only be propagated by vegetative means [7]. Interestingly, the virus isolates from these two ‘Tift94’ accessions only had 96.0% nt sequence identity, which suggests independent acquisition of the virus from local sources following the single introduction of the grass into Australia. ‘DT-1’ is also a sterile hybrid Bermuda grass cultivar imported as a single introduction from the USA [14]. Two independent samples of this grass were tested for BGLV, only one of which was infected. This result again points to relatively recent spread of the virus into this cultivar, after import of the cultivar into Australia.

Symptoms were not observed in any of the eight Bermuda grass accessions with BGLV infection in the glasshouse until July 2021, more than two years after the plants were first established. At this time, mild mosaic symptoms were observed on the leaves of the ‘Gully Gold’ plant (isolate 5663; Fig. 1b). No other plant viruses that are known to infect turf grasses were detected in this plant by transmission electron microscopy, RT-PCR using primers U341/D341 for potyviruses in general [10], primers Grassnepo-F1/Grassnepo-R1 for Stenotaphrum nepovirus [18], or primers PMV-F1/PMV-R1 for Panicum mosaic virus [4], suggesting that the symptoms were associated with BGLV. We propose that the symptoms of BGLV infection are transient in expression and are affected by the stage of plant growth, as well as the prevailing environmental conditions. It is also possible that BGLV supports the replication of satellite viruses or RNAs that modulate symptoms, as with PMV [13]. Further research is required to investigate the pathogenicity of BGLV, ideally using an infectious clone.

Finally, a Rhodes grass plant (virus isolate 5651, Table 1) with strong mosaic symptoms (Fig. 1c) was collected from Oxley Creek Common, a public park in the Brisbane suburb of Rocklea. Identification of the plant species was confirmed by *matK* gene sequencing as described above, and the sequence was deposited in the GenBank database (Table 1). This particular plant was growing in a mixed sward of pasture grasses that was regularly mown to maintain public access. When a sap extract was viewed under a JEOL JEM-1400 electron microscope, two types of virion were observed, one flexuous and filamentous, 700–750 × 10–11 nm, and the other, isometric with a modal diameter of 27 nm (Fig. 1e). RT-PCR was done using the universal potyvirus primer pair U341/D341 [10] and a *c*. 340-nt amplicon was obtained and sequenced (GenBank OK258313). The closest match (95.8% nt sequence identity over the entire length of the amplicon sequence) was to an SCMV isolate from St. Augustine grass found on a turf farm in New South Wales (GenBank MW026606). A universal panicovirus RT-PCR was also done, and again, the plant tested positive, with amplicon sequencing confirming infection by BGLV. Pairwise sequence comparisons showed that this virus isolate has 87.4% nt sequence identity to the exemplar isolate of BGLV and 96.7% identity to the virus isolate in ‘Gully Gold’ (isolate 5663) from New South Wales (Supplementary Table S2).

It was hypothesised that the BGLV isolate found in Rhodes grass from Oxley Creek Common originated from Bermuda grass plants that are also present there. Surveys were done in August 2021, and eight of the 11 plants that were sampled from within a *c*. 15-m radius of the original infected Rhodes grass plant were RT-PCR positive using the universal panicovirus assay. Three of the eight BGLV-positive Bermuda grass samples were lodged in the QDAF Plant Virus Collection under virus isolate numbers 5812, 5815, and 5816 (Table 1). Identifications of the hosts were confirmed by *matK* gene sequencing as described above, and the sequences were deposited in the GenBank database (Table 1). Direct amplicon sequencing was done, and all three virus isolates had >99.0% nt sequence identity to each other and to the BGLV isolate from Rhodes grass and only 96.7-97.0% nt sequence identity to the next most closely related isolate from Bermuda grass ‘Gully Gold’ (isolate 5663) from New South Wales (Supplementary Table S2). This result does suggest a common origin of the BGLV isolates from the park. However, even within this relatively small area, sequence variation was observed, as the three virus isolates from Bermuda grass and the single virus isolate from Rhodes grass all differed from each other by one to three single nucleotide polymorphisms.

Kikuyu grass was also common at Oxley Creek Common in the vicinity of the original infected Rhodes grass plant, and four asymptomatic plants were sampled randomly. Two of these four plants tested positive for panicovirus by RT-PCR. The two samples were lodged in the QDAF Plant Virus Collection under virus isolate numbers 5813 and 5814 with their *matK* gene sequences deposited in the GenBank database (Table 1). Sequencing of the panicovirus RT-PCR amplicon confirmed the presence of BGLV, and these virus isolates had >99.0% nt sequence identity to the other virus isolates from the park (Supplementary Table S2). Mirroring previous results, the amplicon sequences for the two virus isolates from Kikuyu grass were non-identical (99.7% nt sequence identity) (Supplementary Table S2).

A phylogenetic analysis was done based on a BGLV amplicon sequence alignment, excluding the primers, using the maximum-likelihood (ML) search method implemented in the RA×ML plugin in Geneious Prime. The GTR GAMMA nucleotide model was selected, with rapid bootstrapping of 1000 replicates, searches for the best-scoring ML tree algorithm, and starting from a random tree topology. The phylogenetic tree grouped all Australian isolates into the BGLV clade (Fig. 2). The Oxley Creek Common group of virus isolates formed a distinct clade that also contained the virus isolate 5663 from ‘Gully Gold’ from New South Wales (Fig. 2).

**Fig. 2:**
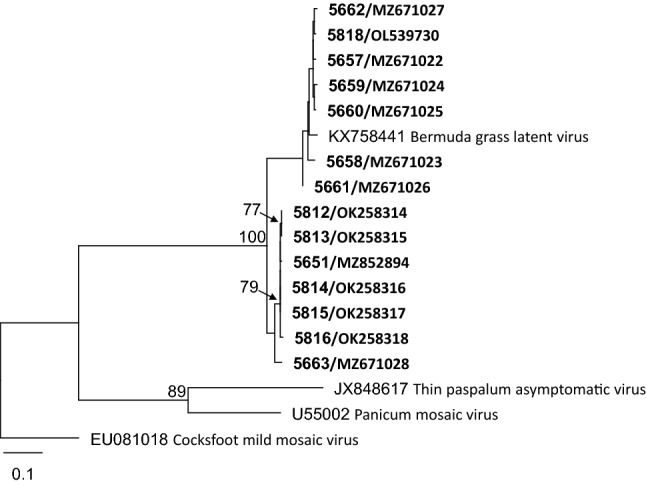
Unrooted maximum-likelihood phylogenetic tree showing the relationships between the various Bermuda grass latent virus isolates (in bold font, listed as isolate number/GenBank accession number) and members of the genus *Panicovirus* based on a nucleotide sequence alignment of a portion of the region encoding the RNA-dependent RNA polymerase. The reference sequences are of the species exemplar isolates. Bootstrap values greater than 70% are shown at the nodes of the branches. The scale bar represents the number of substitutions per site

In this study, we report for the first time the presence of BGLV in Australia, which represents only the second record of this virus anywhere in the world after the USA. BGLV was also detected in two hybrid Bermuda grass cultivars, as well as Rhodes grass and Kikuyu grass, representing extensions to the natural host range of this virus. Given the relative ease with which BGLV was found in New South Wales and Queensland using the RT-PCR diagnostic tool described in this paper, it is likely that BGLV occurs throughout Australia and in many more countries around the world, wherever Bermuda grass occurs. Turf grass species are normally traded as vegetative propagules in order to maintain genetic purity and also because many cultivars have poor fertility. Hence, turf grasses probably represent an important pathway for the spread of BGLV, as well as a range of other grass-infecting viruses.

Our finding of a severely diseased plant of Rhodes grass that was infected by both SCMV and BGLV raises the question of synergism between these two viruses, similar to that proposed to occur in St. Augustine grass in Florida, resulting in lethal viral necrosis [8]. Investigating the host range within turfgrass and cereal crop species, the method of transmission of BGLV, and potential synergistic interactions between this virus and SCMV should be an urgent research priority, especially for the Australian turfgrass industry.

## Supplementary Information

Below is the link to the electronic supplementary material.

Supplementary file1 Figure S1 Genome organization of Bermuda grass latent virus isolate 5657 showing five predicted open reading frames (orange boxes) and the putative protein products (green boxes). RT = internal amber stop codon that can be read through. The scale is the number of nucleotides (PPTX 802 KB)

Supplementary file2 (TXT 17 KB)

Supplementary file3 (DOCX 32 KB)

Supplementary file4 (XLSX 21 KB)

## Data Availability

The sequence data that support the findings of this study have been deposited in the GenBank database with accession numbers provided in the manuscript.
