# Supplementary material for: Bermuda grass latent virus in Australia: genome sequence, sequence variation, and new hosts
Source: Arch Virol. 2022 Apr 8;167(5):1317–23. doi: 10.1007/s00705-022-05434-6 (PMC9038842; doi:10.1007/s00705-022-05434-6)
Supplement: Supplementary file 1 — Supplementary file1 Figure S1 Genome organization of Bermuda grass latent virus isolate 5657 showing five predicted open reading frames (orange boxes) and the putative protein products (green boxes). RT = internal amber stop codon that can be read through. The scale is the number of nucleotides (PPTX 802 KB) [file 705_2022_5434_MOESM1_ESM.pptx]

## Slide 1
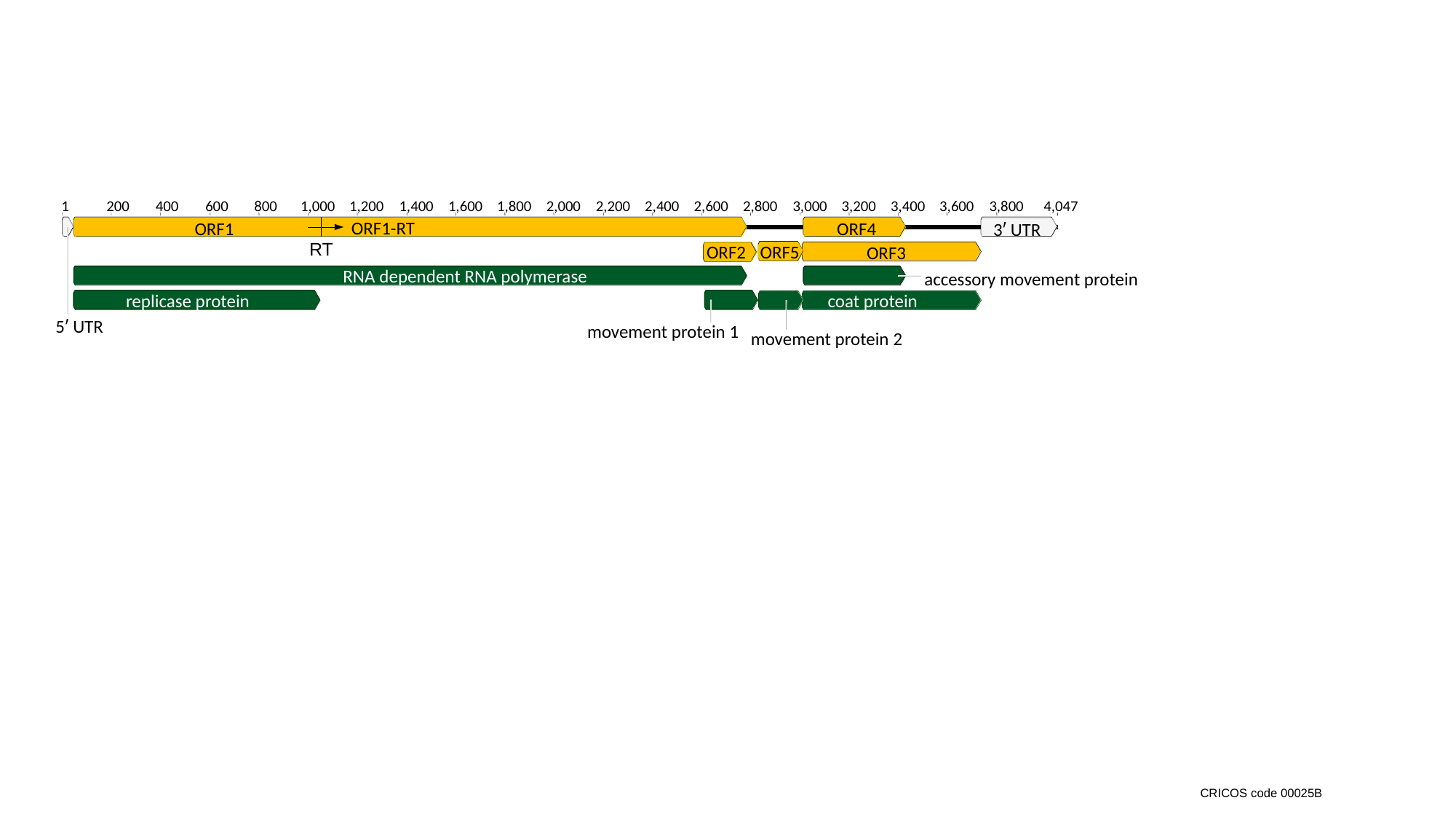

1
200
400
600
800
1,000
1,200
1,400
1,600
1,800
2,000
2,200
2,400
2,600
2,800
3,000
3,200
3,400
3,600
3,800
4,047
ORF4
ORF1
3ʹ UTR
ORF2
ORF5
ORF3
RNA dependent RNA polymerase
accessory movement protein
coat protein
replicase protein
5ʹ UTR
movement protein 1
movement protein 2
ORF1-RT
RT
