# Supplementary material for: Bermuda grass latent virus in Australia: genome sequence, sequence variation, and new hosts
Source: Arch Virol. 2022 Apr 8;167(5):1317–23. doi: 10.1007/s00705-022-05434-6 (PMC9038842; doi:10.1007/s00705-022-05434-6)
Supplement: Supplementary file 3 — Supplementary file3 (DOCX 32 KB) [file 705_2022_5434_MOESM3_ESM.docx]

**Supplementary Table S1** Virus specific primers used in 5ʹ/3ʹ Rapid Amplification of cDNA Ends (RACE) for Bermuda grass latent virus isolate 5657

| **Experiment** | **Primer name** | **Sequence 5ʹ to 3ʹ** | **Usage** |
| --- | --- | --- | --- |
| 5ʹ RACE | BGLV-SP1-R | GTAGATGCTAGCAGCGGTTAGG | cDNA synthesis |
|  | BGLV-SP2-R | TGGATCTGTCCTAGCGGGTCAAC | PCR and Sanger sequencing |
|  | BGLV-SP3-R | CCTCTCGATAGACTCACATTTAAGC | Nested Sanger sequencing |
| 3ʹ RACE | BGLV-SP5-F1 | CGTGAGCTAGGATGGCTATCCTATC | PCR and Sanger sequencing |
|  | BGLV-3UTR-F1 | TCCATTAGCGCTGTGGTGTC | Nested Sanger sequencing |
